# Experimental study of impact mechanical and microstructural properties of modified carbon fiber reinforced concrete

**DOI:** 10.1038/s41598-022-17092-4

**Published:** 2022-07-28

**Authors:** Yuhang Du, Song Lu, Jinyu Xu, Wei Xia, Tengjiao Wang, Zhihang Wang

**Affiliations:** 1 Aviation Engineering School, Air Force Engineering University, Xi’an, 710038 China; 2 College of Mechanics and Civil Architecture, Northwestern Polytechnical University, Xi’an, 710072 China

**Keywords:** Engineering, Materials science

## Abstract

This paper investigated the preparation method and the dispersion behaviour of Modified Carbon Nanotube-fiber Reinforcements (MCNF), the change laws and the effect mechanisms of dynamic compressive strength of MCNF concretes. Electrophoresis method was used to prepare MCNF and its interfacial shear performance was tested by interfacial shear strength (IFSS) test. In addition, the dispersion behavior of MCNF in simulated concrete solution was verified by turbidity method. Split Hopkinson Pressure Bar (SHPB), Scanning Electron Microscope (SEM) and Mercury Intrusion Porosimetry (MIP) tests were carried on concrete samples with different volume fractions (0%, 0.1%, 0.2%, 0.3%, 0.4%) of MCNF. The results show that carbon nanotubes are easier to deposit to the negative electrode, and the higher the content of polycarboxylate superplasticizer, the more obvious the dispersity of MCNF in alkaline environment. The dynamic compressive strength of MCNF concrete was 14.0–35.5% higher than that of untreated concrete, and reached the maximum when the MCNF content was about 0.3%. The MCNF was wrapped in concrete matrix and promoted hydration reaction of interface between cement and MCNF from microscopic observation. The addition of MCNF could increase the porosity. The volume percentage of ≥ 100 nm pore decreased first and then increased. Reasons for the improvement strength of MCNF concrete is that the bridging effect is stronger with the increase of MCNF content (≤ 0.3%) and limited when the MCNF content is equal to 0.4%. MCNF concrete could be used in actual engineering with high requirements for dynamic load.

## Introduction

Nearly all construction buildings suffer dynamic loads, such as wind load, earthquake load and violent collision. The impact load of weapon explosion has been considered in structural design of military protection facilities, underground command posts and other national defense projects. Concrete is the primary material used in the design and construction of civil architecture and national defense engineering. Therefore, how to effectively improve the dynamic impact mechanical properties of concrete has become the focus and hotspot of the research and development of concrete materials. In this regard, carbon fiber, steel fiber and synthetic fiber are traditionally used to improve the tensile strength and ductility of concrete^1^. On the other hand, scholars have carried out extensive studies on the mechanical properties of various fiber reinforced concrete, such as steel fiber reinforced concrete^2–7^, basalt fiber reinforced concrete^8–10^, carbon fiber reinforced concrete^11–16^, and polyethylene fiber reinforced concrete^17,18^. Fiber-modified concrete has become one of the development trends of concrete in the future.

Comparatively speaking, carbon fiber and steel fiber reinforced concrete are the most widely studied and used at present. Though the effect of steel fiber on the mechanical properties of concrete is obvious, the utilization of steel fiber is limited to a certain extent due to its high self-weight and corrosion tendency. In contrast, carbon fiber not only has the advantages of high tensile strength, large elastic modulus and strong toughness, but also has excellent electrical and thermal conductivity, wear resistance and corrosion resistance. Taking the advantages of its high strength, light weight, corrosion resistance, fatigue resistance and excellent high-temperature stability, when mixed into concrete, it can not only enhance the strength and resist cracking, but also endow concrete with intelligent functions, such as electric conductivity, pressure sensitivity, temperature sensitivity, and electromagnetic shielding^19^. Therefore, it is widely used by the civil engineering community. This paper found that the traditional carbon fiber reinforced concrete has some shortcomings, such as poor surface interfacial properties and weak adhesion with the cement matrix. Under heavy loads, carbon fiber and concrete matrix are prone to slip failure, which affects the macroscopic mechanical properties of carbon fiber reinforced concrete, and has become one of the main factors restricting the development of carbon fiber reinforced concrete.

MCNF is a kind of carbon fiber material. Carbon nanotubes have a unique one-dimensional tubular structure with high strength and toughness. Its Young's modulus is estimated to be 5 TPa theoretically, and the measured value is 1.8 TPa through laboratory tests, besides, its tensile strength is 100 times that of ordinary steel^20^. The combination of carbon fibers and carbon nanotubes can effectively overcome the shortcomings of poor surface interfacial properties and weak adhesion with the cement matrix, making them a hotspot in the field of novel material research^21^. The current studies on the preparation of MCNF are mature, and the main preparation methods include chemical grafting^22^, electrophoretic deposition^23^, CVD^24^, sizing^25^, etc. MCNF is currently still mainly used for research, which is seldom applied, let alone its application in concrete. And the research on the explosive impact resistance of concrete is even rarer. Due to its strain rate sensitivity, the mechanical properties of concrete material under dynamic loads differ from those under static loads^26^. Therefore, it is necessary to study the dynamic mechanics of MCNF concrete.

MCNF was prepared by using electrophoretic deposition method, and its interfacial shear performance was tested by interfacial shear strength test. The dispersion ability of MCNF was verified based on turbidimeter method. In addition, the effect of MCNF content and impact velocity on the failure patterns, dynamic compressive strengths of MCNF concrete was studied through SHPB tests. The microstructure and pore distribution of MCNF concrete were examined by SEM and MIP tests separately. And the modification mechanisms of MCNF concrete were deeply analyzed. All results are of theoretical significance for engineering design and construction with high requirements for dynamic load.

## Materials and methods

### Test materials

The carbon fiber used (T700SC-1000-50C) and the carbon nanotube (XFM06) were taken from Toray Industries of Japan and Nanjing Xianfeng Nanomaterials Technology Co. Ltd. of China, respectively. The main parameters of both products are shown in Tables 1 and 2, respectively. The cement used (P.O 42.5) and polycarboxylic water reducer of concrete were obtained from Conch Cement Co., Ltd. and Hunan Zhongyan Technology Co., Ltd. of China, respectively. The used sands and stones of concrete were from Bahe River of China, and the sands were in medium size with the fineness modulus of 2.7.

**Table 1:** Main parameters of carbon fiber.

Tensile strength	Tensile modulus	Elongation value	Density	Diameter	Sizing agent
4900 Mpa	230 Gpa	2.1%	1.76 g/cm^3^	7 μm	50C 1.0%

**Table 2:** Main parameters of carbon nanotubes.

Outer diameter	Length	Purity	Carboxylated content	Specific surface area	Tap density
5–15 nm	0.5–2 μm	≥ 95%	3.86 g/cm^3^	> 200 m^2^/g	0.27 g/cm^3^

### Test Methods

#### Preparation and verification of MCNF

MCNF is prepared by electrophoresis. Electrophoresis refers to the phenomenon that the charged particles move towards the electrode opposite to their electrical properties under the action of electric field. The electrophoresis equipment used is composed of a DYY-8C power supply from Beijing LIUYI Biotechnology Co., Ltd. and a self-made electrophoresis tank. The turbidimeter used (SGZ-200BS) is produced by Shanghai Yuefeng Instrument Co., LTD. of China.

The specific preparation process of MCNF is described as follows. Firstly, remove surface sizing of carbon fibers by soaking them in acetone for 48 h, and then clean them in 200 ml alcohol for three times and in 200 ml deionized water for another three times. Dry them to constant weight at 60 ℃ before cooling them down to room temperature. Add carbon nanotubes (0.375 g) to cetyl trimethyl ammonium bromide (CTAB) solutions (0.00092 mol/l, 250 ml) under the dispersion by ultrasonic waves for 2 h. The electrophoresis tests were conducted with carbon fiber as the positive or negative electrode, and the stain less steel sheet as the other electrode. The voltage was set to 60 V, and the electrophoresis duration was 1 h^23^.

Turbidity is used to indicate the dispersion degree of MCNF. It refers to the obstruction degree of the solution to the passage of light, including the scattering of light by suspended solids and the absorption of light by solute molecules. The specific verification process of dispersive behaviour of MCNF is described as follows. Add MCNFs (6 mm long) and polycarboxylic water reducers (20 ml, set concentration) into saturated calcium hydroxide (10 ml), and oscillate the mixture for 3 Min. by magnetic oscillator equipment. Finally, the turbidity of mix solution was obtained by turbidimeter^27^.

#### Interfacial shear strength (IFSS) test

**Figure 1:**
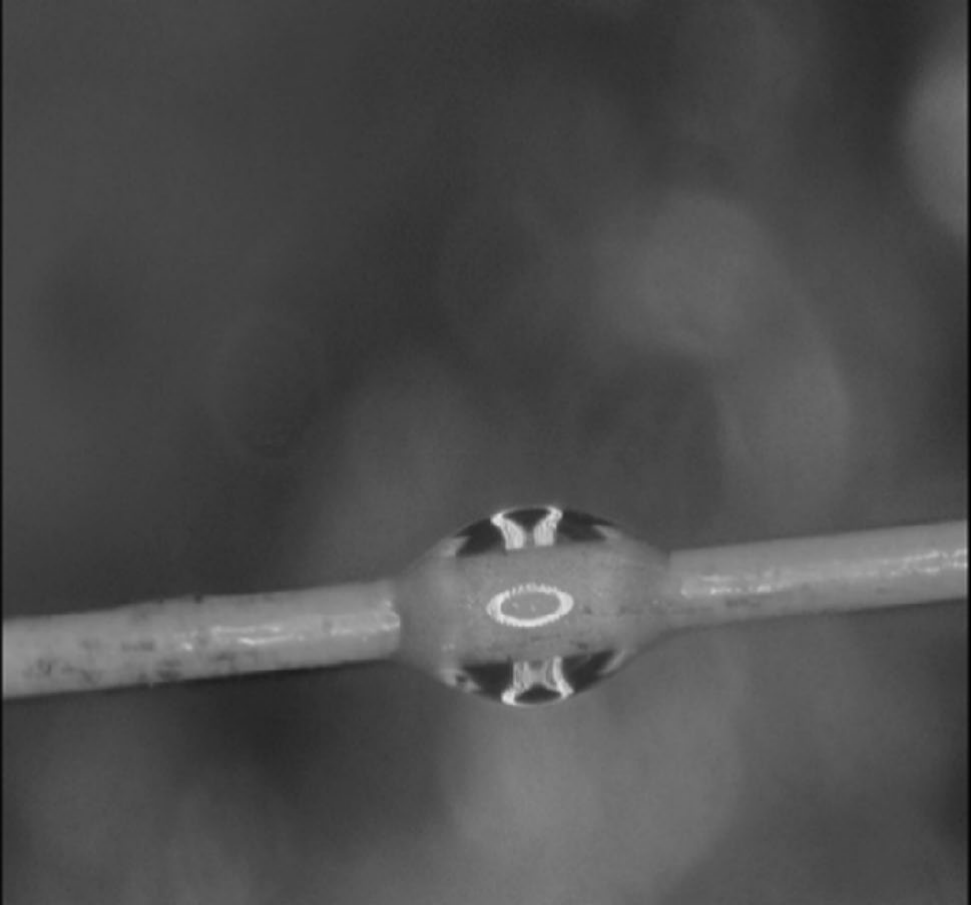
Monofilament droplet debonding test chart.

In order to verify the preparation effect of MCNF better, the interfacial shear strength test of carbon fiber and MCNF was carried out. First, a single carbon fiber was taken out from the carbon fiber bundle, fixed on a concave metal plate with double-sided tape, and then the uncured polymer matrix was slightly contacted with the fiber surface with a metal needle. The polymer matrix forms oval droplets on the fiber surface, as shown in Fig. 1.

After the droplet matrix was completely solidified, the metal sheet was fixed in the center of a pair of symmetrical blades of the interfacial strength evolution instrument, and then a uniform load was applied to one end of the fiber until the fiber was pulled out from the droplet, and the pulling force-time curve was recorded. The fiber is pulled out, and the maximum tensile load during the pull-out process is obtained. The interface bond strength value between the monofilament carbon fiber and the resin matrix can be calculated according to the following formula:

1 $$ IFSS = \frac{F\max }{{\pi dfLe}} $$

where *IFSS* refers to the interfacial shear strength, *F*_*max*_ refers to the maximum load when the carbon fiber is peeled off, *d*_*f*_ refers to the diameter of the carbon fiber, *L*_*e*_ refers to the length of carbon fiber wrapped in vertical ball.

#### Preparation of MCNF concrete

The mix ratio of concrete is shown in Table 3. Firstly, mix sands and stones, and add cements. Secondly, add a set amount of MCNFs. Finally, keep stirring until the materials were well mixed, and then pour the mixture into the custom molds for vibration molding. Standard curing was adopted for the demoulded samples after 24 h., and precision machining was conducted with grinder to achieve the surface roughness below 0.02 mm after standard curing.

**Table 3:** The mix ratio of concrete (kg/m^3^).

Cement	Sand	Pebble	Water	Water reducing	MCNF			
					0.1%	0.2%	0.3%	0.4%
340	640	1360	130	1.7	1.76	3.52	5.28	7.04

#### SHPB tests of MCNF concrete

The concrete samples for SHPB tests are cylinders in the size of Φ 98 mm × 48 mm. To eliminate the interface friction effect, the mixture of graphite and lubricant in the shape of an even thin layer was applied on both ends of concrete samples before the tests.

**Figure 2:**
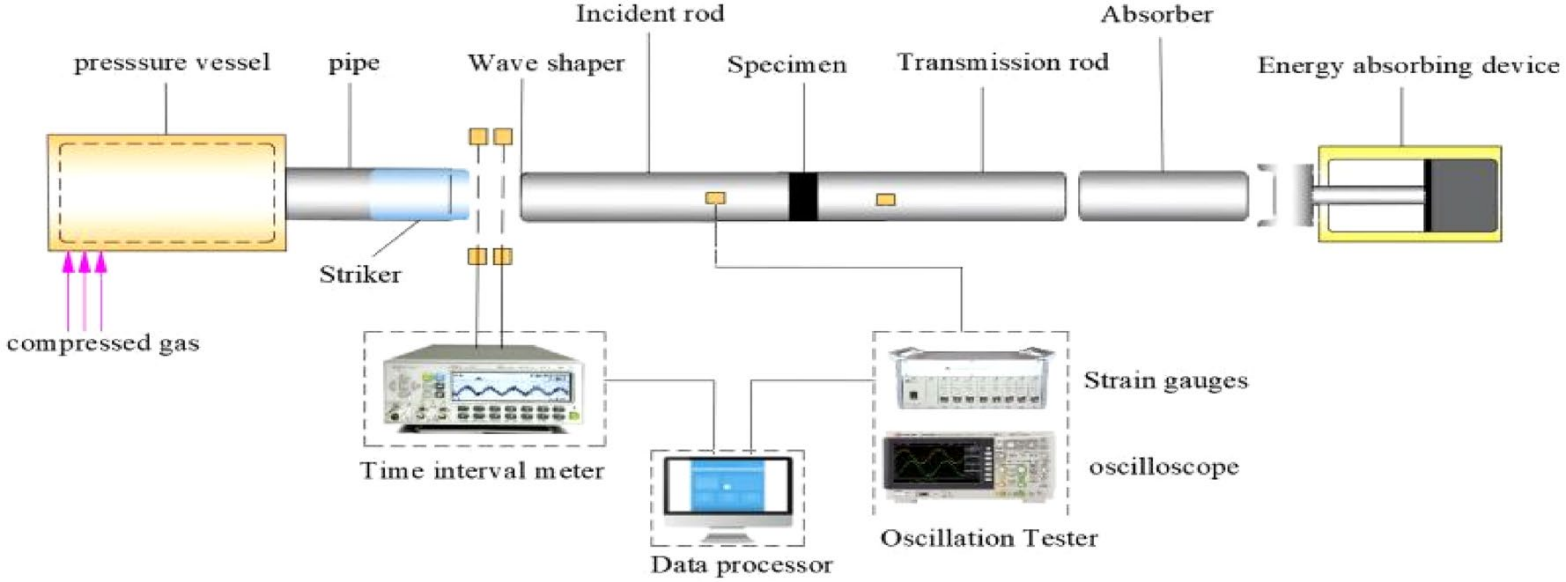
Schematic diagram of SHPB equipment.

The schematic diagram of φ100 mm SHPB equipment is shown in Fig. 2, including the main equipment, energy systems and test systems. The main equipment consists of transmission rod, incident rod and striker. The energy system is composed of energy absorbing device, gas line and gas tank. And the test systems include time interval mater, data processor, strain gauges, oscilloscope and SHPB main equipment.

The SHPB tests are based on the elastic stress propagation theory in slender rods. It has two basic assumptions, i.e., the plane assumption and the uniform stress assumption. The former is that each cross section of the elastic rod stays in plane state when the stress wave propagates in the slender rod. And the latter is that the stress is equal everywhere inside the sample^28^.

Three-wave method^29^ was adopted to calculate the stress, strain rate and strain according to the stress waveform in the elastic rod. The formulas are expressed as follows.

2 $$ \varepsilon_{s} (t) = \frac{{C_{e} }}{L}\int_{0}^{\tau } {[\varepsilon_{I} (t) - \varepsilon_{R} (t) - \varepsilon_{T} (t)]dt} $$

3 $$ \overline{\dot{\varepsilon }}_{s} \left( t \right) = \frac{{C_{e} }}{L}[\varepsilon_{I} (t) - \varepsilon_{R} (t) - \varepsilon_{T} (t)] $$

4 $$ \sigma_{s} (t) = \frac{{E_{e} A_{e} }}{{2A_{s} }}[\varepsilon_{I} (t) + \varepsilon_{R} (t) + \varepsilon_{T} (t)] $$

where *Ce* refers to the velocity of stress wave propagation in the rod,* L* stands for the thickness of sample, *τ* represents the propagation time of stress wave in rod, *Ee* and *Ae* are the elastic modulus and cross section area of rod, respectively, and *As* denotes the area at end face of sample.

#### SEM and MIP tests of MCNF concrete

Select the samples with suitable size after crushing by SHPB tests. After that, remove the residues and dusts from the surface, and then dehydrate them completely. Coated with gold, SEM samples were observed with the appropriate magnification and scope, the others were the examined pore distribution by using Mack AutoPore IV 9500. The mercury infiltration angle was 130°, and the pressure ranged from 20 to 33,000 psi.

### Tests schedule

The detailed test schedule is shown in Table 4. The adsorption effects of carbon fiber as positive and negative electrodes on carbon nanotubes were discussed respectively based on electrophoresis tests, The interfacial shear strength of MCNF prepared by electrophoresis and carbon fiber were tested. The effect law of different water reducer contents (0, 0.5%, 1.0%, and 1.5%) on the dispersion degree of 0.1% MCNF was obtained through turbidity tests. The variables of SPHB tests include impact velocity (6.8 m/s, 7.8 m/s, 8.8 m/s, 9.8 m/s, and 10.8 m/s) and MCNF content (0%, 0.1%, 0.2%, 0.3%, and 0.4%). The concrete samples crushed in SHPB tests with the content of MCNF of 0 and 0.4%, respectively were observed by using SEM equipment. The MIP tests mainly considered the influence of different MCNF contents (0%, 0.1%, 0.2%, 0.3%, 0.4%) on pore structure.

**Table 4:** The specific tests schedule.

Tests	Electrodes	Water reducer content (%)	MCNF content (%)	Impact velocity (m/s)
Electrophoresis	+, −	–	–	–
IFSS testing	–	–	–	–
Turbidity	–	0, 0.5, 1.0, 1.5	0.2	–
SHPB	–	–	0, 0.1, 0.2, 0.3, 0.4	6.8, 7.8, 8.8, 9.8, 10.8
SEM	–	–	0, 0.4	–
MIP	–	–	0, 0.1, 0.2, 0.3, 0.4	–

## Results and discussion

### Adsorption effect of carbon nanotubes on carbon fibers

**Figure 3:**
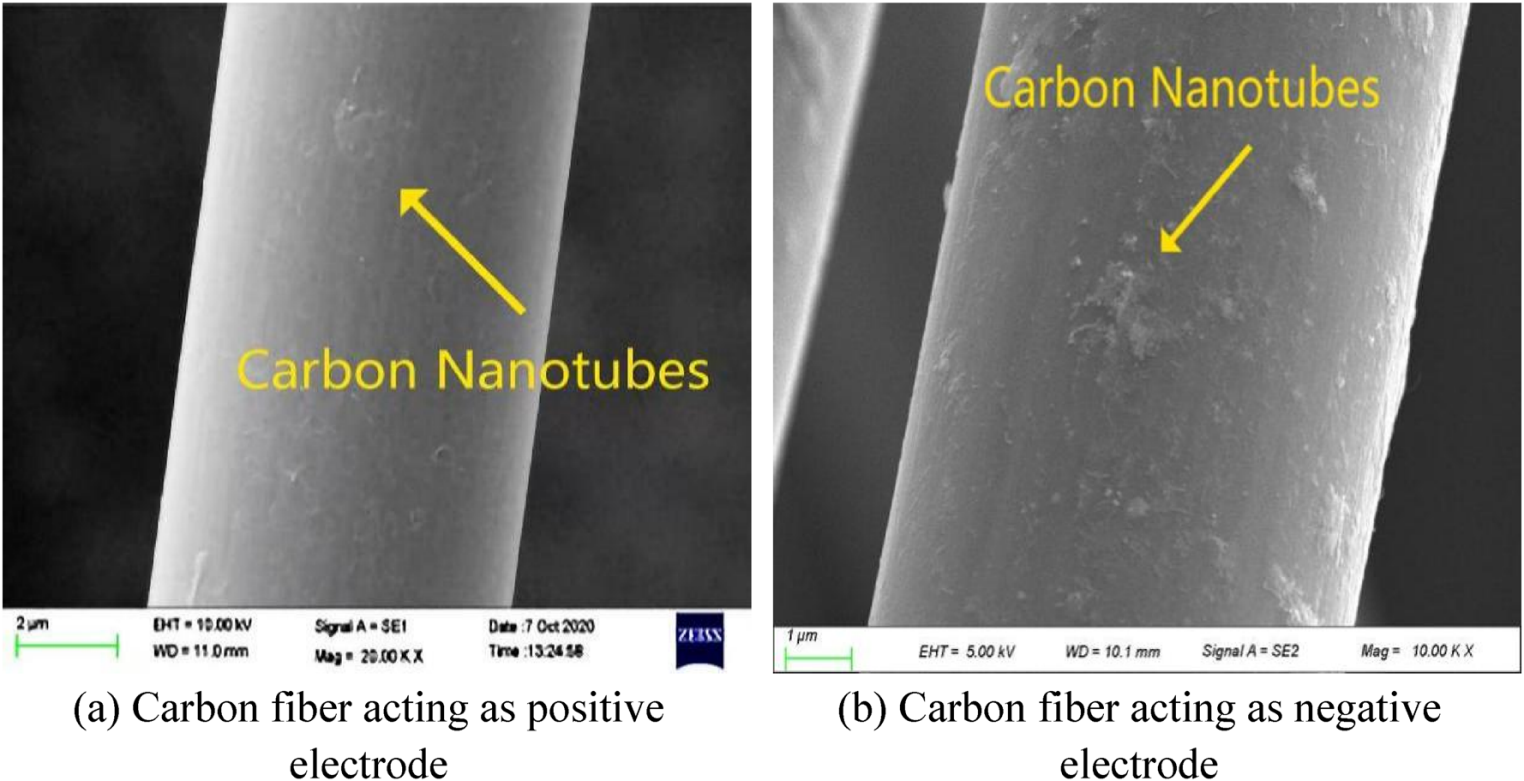
Micro pictures of MCNF using carbon fiber as positive electrode and negative electrode.

It can be seen from the electrophoresis test results that carbon nanotubes were deposited regardless of whether the carbon fiber was positive electrode or negative electrode with the stain less steel sheet taken as another electrode in the same CTAB solution environment. It is similar to the research result of Guo Jinhai^30^. However, the deposition amount of carbon nanotubes was much higher when carbon fiber was used as negative electrode. The micro pictures of MCNF using carbon fiber as negative electrode and negative electrode are shown in Fig. 3. It is obvious that the surface of carbon fiber acting as negative electrode was rougher when compared with that as positive one. Therefore, it is suggested to use stainless steel sheet as the positive electrode and carbon fiber as the negative one to for the preparation of MCNF materials.

The reason for the above phenomenon is that when using CTAB as the dispersion solution, based on the polarity of water and the non-polarity of carbon nanotubes, the surfactant molecules on the interface, namely the surface of carbon nanotubes, are arranged in order. The polar end of the surfactant is in water, which is positively charged, while the non-polar end is on the surface of the carbon nanotubes, therefore, the carbon nanotubes are positively charged in CTAB, and deposited onto the negative electrode.

### Interfacial shear strength test results of MCNF and carbon fiber

The interfacial shear strength test results is shown in Table 5, which showed that the interfacial shear strength of MCNF was significantly stronger than that of carbon fibers. Compared with the interfacial shear strength of carbon fibers, the interfacial shear strength of MCNF was increased by about 40.8%, The carbon nanotubes of the MCNF make the MCNF surface rougher than carbon fiber, so the pullout resistance is better.

**Table 5:** IFSS of various single fibers droplet composites.

Fiber type	Interfacial shear strength (MPa)	Improvement (%)
Carbon fiber	53.2	–
MCNF	74.9	40.8

### Turbidity of MCNF in simulated concrete solution

**Figure 4:**
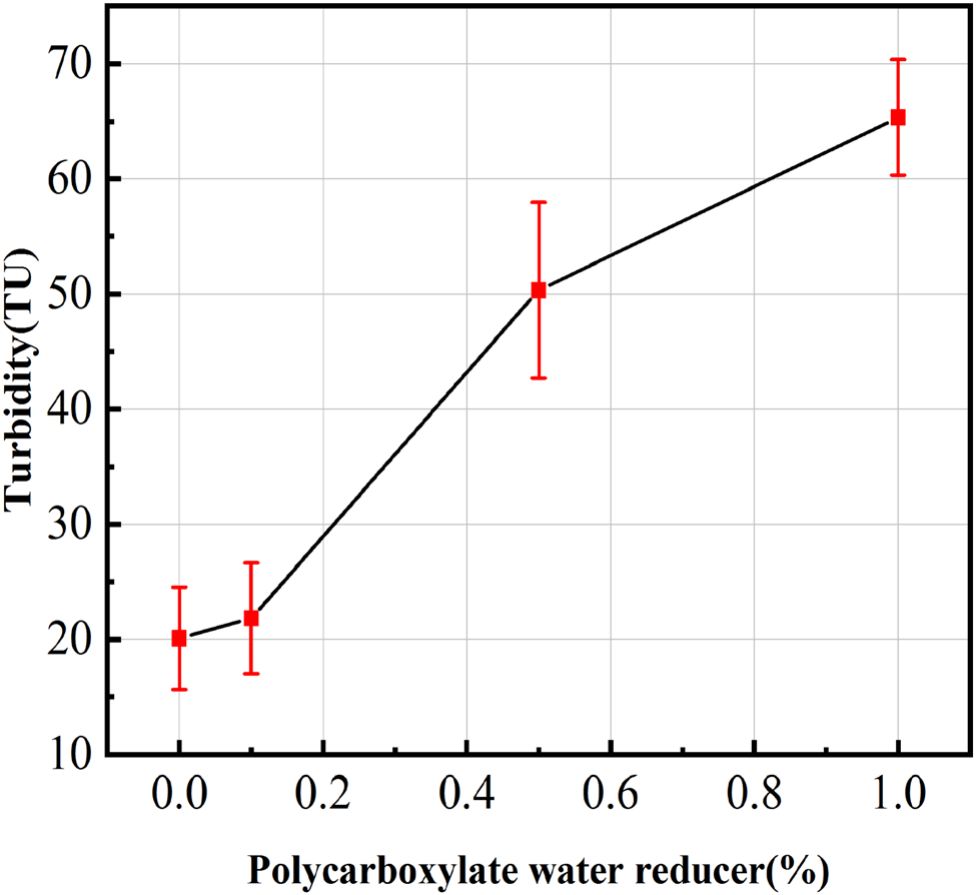
Relationship between turbidity and polycarboxylate water reducer.

The result of turbidity tests is shown in Fig. 4. It is obvious that the turbidity is increased from 20.1 TU to 65 TU with the increase of polycarboxylic water reducer content from 0 to 1.0%, indicating that higher water reducer content could promote the dispersion property of MCNF in saturated calcium hydroxide solution. The reason is that the surface of MCNF adsorbed a large number of hydroxyl, carboxylic and sulfonic groups of polycarboxylic water reducer, resulting in the increase of repulsion between MCNFs^27^. In another word, the higher the content of polycarboxylate superplasticizer, the more obvious the dispersity of MCNF in alkaline environment.

### SHPB tests results of MCNF concrete

**Figure 5:**
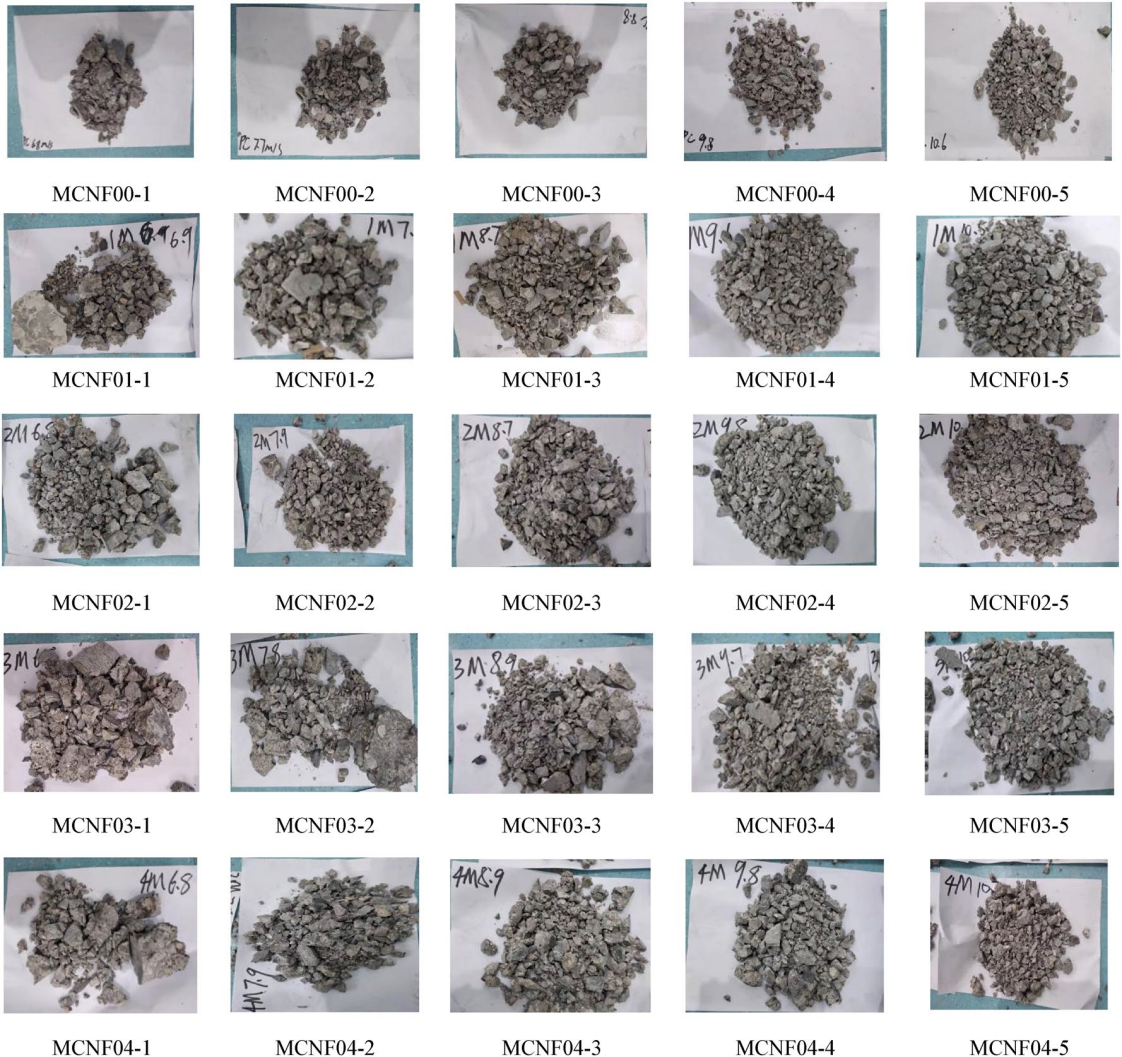
The failure patterns of MCNF concretes under different impact velocity.

**Figure 6:**
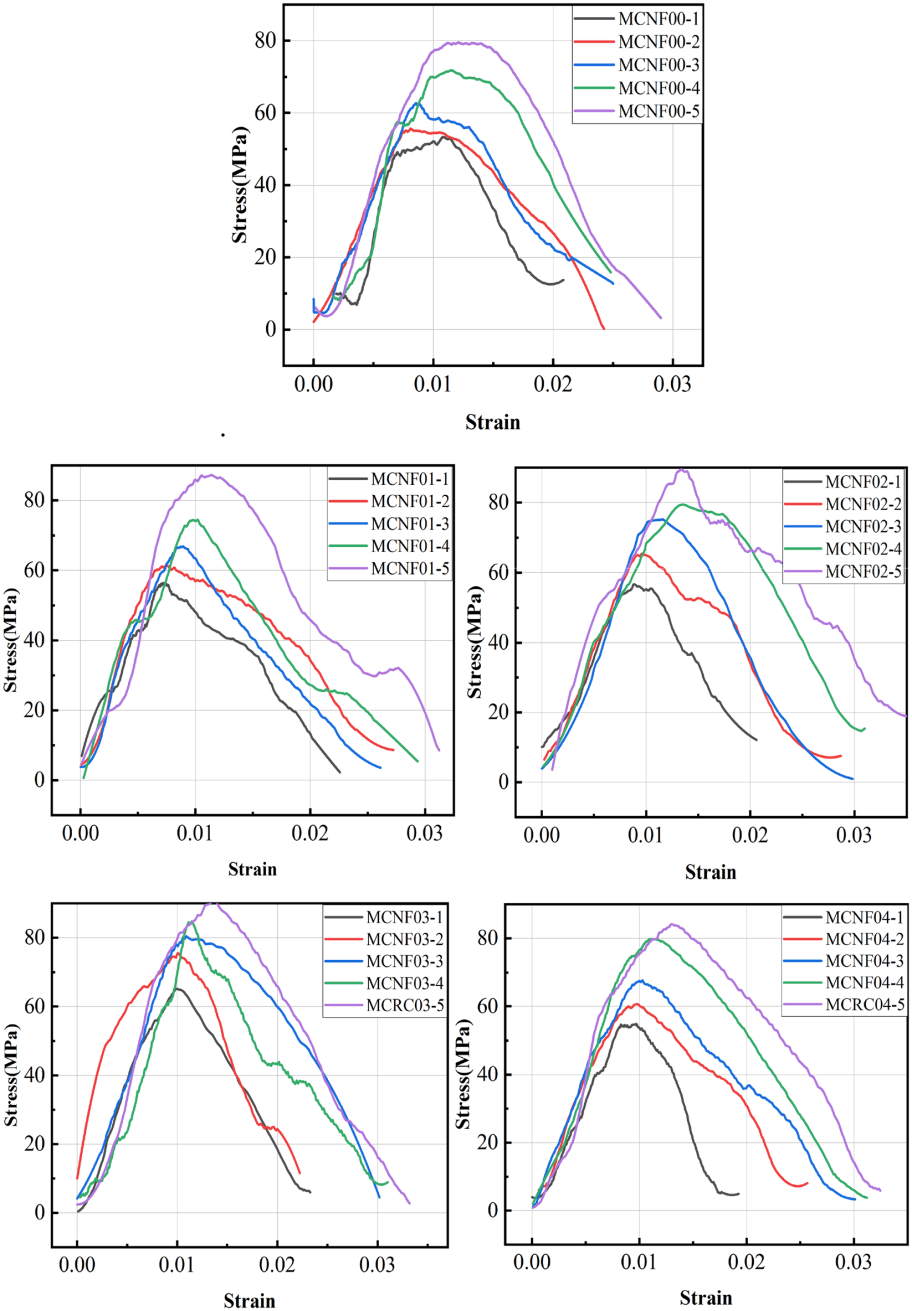
The stress–strain curves of MCNF concretes.

Concrete samples exhibit different failure patterns under different impact forces. The main failure patterns include core retention, spalling, bulk crushing, severe crushing, thorough crushing and so on^31^. The dynamic compressive strength of MCNF concretes under different impact loads is shown in Table 6. And the failure patterns of MCNF concretes at different impact velocity are shown in Fig. 5. Besides, MCNF00, MCNF01, MCNF02, MCNF03 and MCNF04 represent the MCNF concrete with different volume fractions (0%, 0.1%, 0.2%, 0.3%, and 0.4%). It can be seen from Fig. 5 that with the increase of impact speed, the concrete is more shattered, while the concrete with the addition of MCNF at the same impact speed is more intact. However, this phenomenon is not very obvious at too high speed, because the bridging effect can no longer offset the energy brought by too high impact speed. The stress–strain curves of MCNF concretes are shown in Fig. 6.

**Table 6:** SHPB Results of MCNF concretes.

MCNF volume content (%)	Test no.	Impact velocity /*v *(m/s)	Dynamic compressive strength/*f*_*cd*_ (MPa)	Peak strain/*ε*_*p*_ (× 10−^3^)	Ultimate strain/*ε*_*p*_ (× 10−^3^)	Sample failure patterns
0	MCNF00-1	6.8	53.3	10.8	20.8	Bulk crushing
	MCNF00-2	7.7	55.7	8.0	24.2	Bulk crushing
	MCNF00-3	8.8	62.7	8.6	25.0	Severe crushing
	MCNF00-4	9.7	71.7	11.4	24.8	Thorough crushing
	MCNF00-5	10.6	79.5	13.2	29.0	Thorough crushing
0.1	MCNF01-1	6.9	56.4	7.3	22.6	Core retention, spalling
	MCNF01-2	7.9	61.5	7.5	27.2	Bulk crushing
	MCNF01-3	8.7	66.9	8.9	26.1	Severe crushing
	MCNF01-4	9.5	74.5	10.2	29.3	Thorough crushing
	MCNF01-5	10.4	87.3	11.3	31.2	Thorough crushing
0.2	MCNF02-1	6.8	56.7	8.9	20.5	Bulk crushing
	MCNF02-2	7.9	65.4	9.6	28.7	Bulk crushing
	MCNF02-3	8.7	75.2	11.6	29.8	Severe crushing
	MCNF02-4	9.8	79.5	13.5	30.9	Severe crushing
	MCNF02-5	10.6	89.6	13.4	35.3	Thorough crushing
0.3	MCNF03-1	6.8	65.2	9.9	23.2	Bulk crushing
	MCNF03-2	7.8	75.5	10.0	22.2	Core retention, spalling
	MCNF03-3	8.9	80.4	11.0	30.1	Severe crushing
0.3	MCNF03-4	9.7	84.7	11.4	31.0	Severe crushing
	MCNF03-5	10.7	90.6	13.5	33.2	Thorough crushing
0.4	MCNF04-1	6.8	54.8	9.6	19.2	Core retention, spalling
	MCNF04-2	7.9	60.7	9.8	25.7	Bulk crushing
	MCNF04-3	8.8	67.7	10.3	30.1	Bulk crushing
	MCNF04-4	9.8	79.8	11.2	31.2	Severe crushing
	MCNF04-5	10.7	84.2	13.0	32.4	thorough crushing

It can be seen that the geometry of the rising section of the curve of each group of specimens is basically the same, which can be roughly divided into three stages, i.e., compaction stage, linear elastic deformation stage and yield stage. In another word, the initial internal pores of the specimen are compacted under the impact load, and the strength of the specimen increases slowly at this time; when the deformation of the specimen increases linearly to the yield point, and then enters the failure stage, it increases linearly with the strength of the specimen; while when the peak value is reached, the specimen is damaged under compression, and the residual strength decreases gradually.

**Figure 7:**
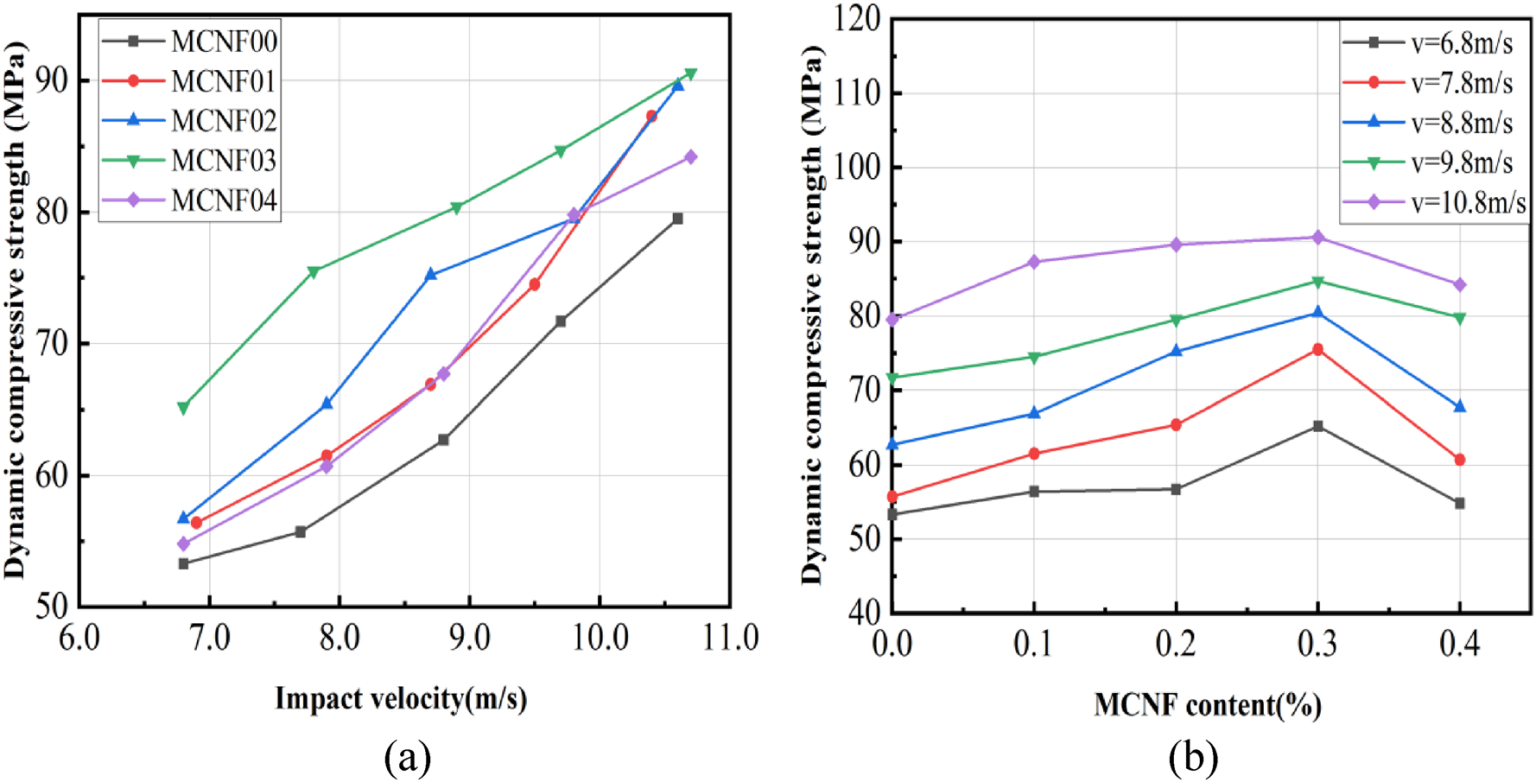
Relationship between impact velocity, MCNF content and dynamic compressive strength.

The effect laws of impact velocity and MCNF content for dynamic compressive strength are shown in Fig. 7.

It can be seen from Fig. 7a that the dynamic compressive strength of concrete increases with the increase of impact velocity. Inevitably, there are many micro-cracks and micro-damages occurred around the aggregates in the concrete. The destruction of concrete materials is mainly due to the extension and development of micro-cracks and micro-damages in the concrete. The energy required to produce new cracks and expand the existing ones is huge, and the former is higher than the latter. Therefore, the higher the impact velocity, the closer the thorough crushing, and the more the cracks expand and generate. In the meantime, concrete fail to absorb the energy through its deformation due to very short impact loading time. According to the functional principle, external energy can only be offset by increasing stress. Therefore, the strength of the concrete increases with the increase of impact velocity. Similar to the analysis of strain rate hardening of rock by Bracc^32^ and Janach^33^ et al., the strain rate hardening effect of concrete can be regarded as the mechanical response of material during the transformation from one-dimensional stress state to one-dimensional strain state. The reason is that the stress inside the sample can no longer be regarded as one-dimensional stress due to the large size in the SHPB test. The surrounding concrete generate hoop pressure to the central concrete. Under the impact load, the concrete will also generate the hoop effect. And the hoop pressure effect is more obvious at larger material size and higher impact velocity^34^. During high-speed impact, the concrete undergoes compression deformation along the impact direction, and the height of the concrete specimen decreases. The diameter increases in the compression direction, and the concrete produces tensile stress. When the tensile stress of the concrete reaches the ultimate tensile strength, the concrete will be damaged.

It can be seen from Fig. 7b that the dynamic mechanical strength of concrete first increased and then decreased with the increase of MCNF content (from 0 to 0.4%) at the same impact velocity, and the threshold was 0.3%, as described in “Tests schedule” section. For carbon fiber reinforced concrete, it is likely to generate stress concentration at both ends of the crack when the concrete is damaged. In contrast, for MCNF concrete, there are many MCNFs in the fibers to share the force, indicating that the crack inhibition effect is more obvious^35^.

### SEM tests results of MCNF concrete

**Figure 8:**
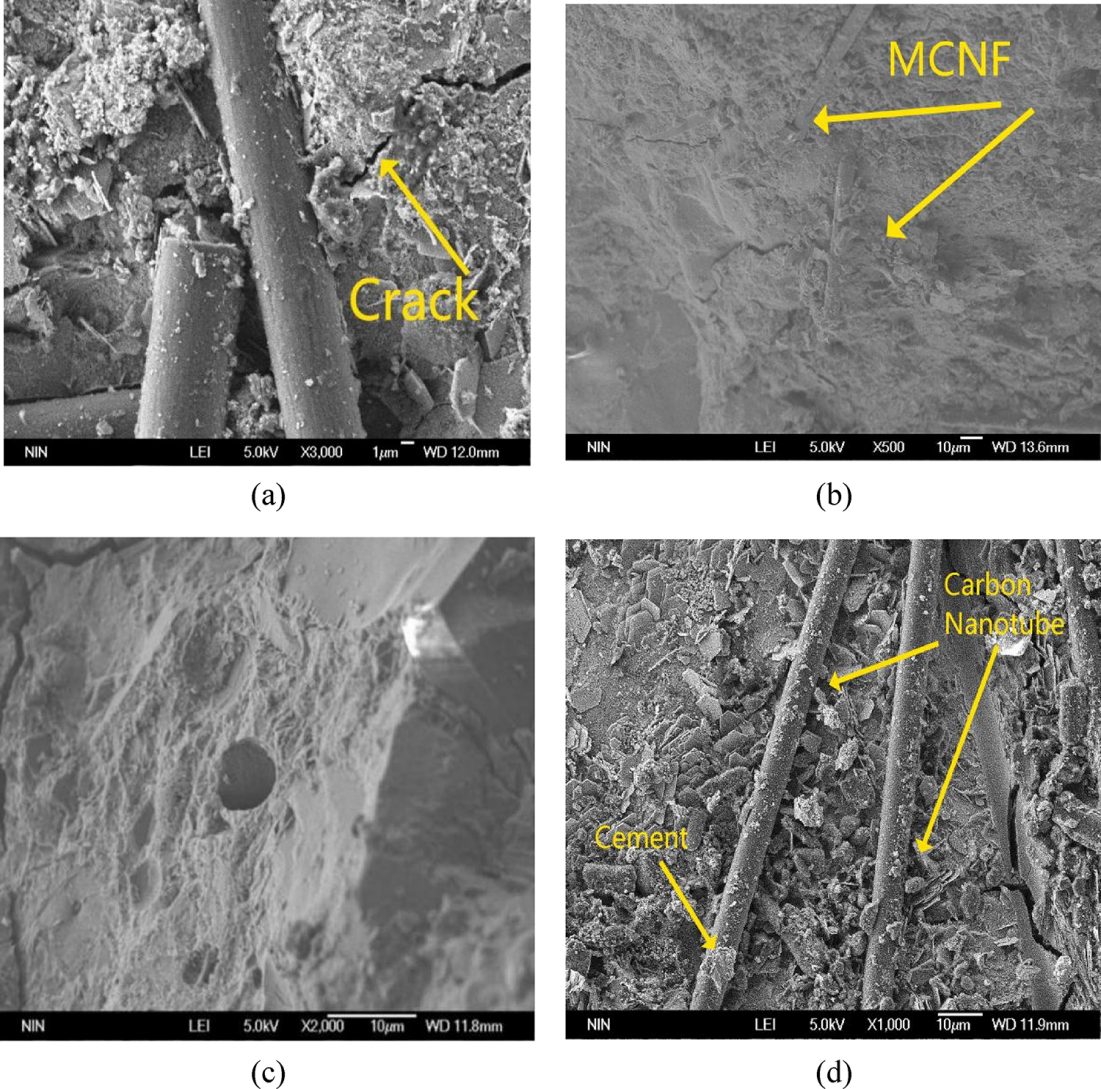
SEM pictures of fracture surface of MCNF concrete.

Micro morphology pictures of concrete with 0.4% MCNF are shown in Fig. 8. Specifically, as shown in Fig. 8a, two exposed MCNFs on the fracture surface of MCNF concrete prevent the development of crack. These carbon nanotubes with strong water absorption characteristics on the surface of carbon fiber resulted in more sufficient hydration reaction of concrete, as shown in Fig. 8b. And MCNF could be observed on the fracture surface, which was wrapped by the concrete matrix. Due to the smooth surface of carbon fiber, it can be easily pulled out under strong load. Figure 8c shows one typical hole left in carbon fiber concrete when carbon fiber was pulled out. In the meantime, Fig. 8d shows the SEM photo of MCNF after being pulled out.It can be clearly seen that carbon nanotubes and cement remain on MCNF, which shows that MCNF is more difficult to pull out than ordinary carbon fibers when pulling out, and even some cement will be brought out. The force required to break inside the cement is much higher than the force required to break the carbon fiber-cement interface. Therefore, MCNF has better pull-out resistance compared to ordinary carbon fibers. In summary, MCNF has better resistant to pull-out, and MCNF concrete could bear stronger impact load with better crack resistance.

### MIP tests results of MCNF concrete and modification mechanism

**Figure 9:**
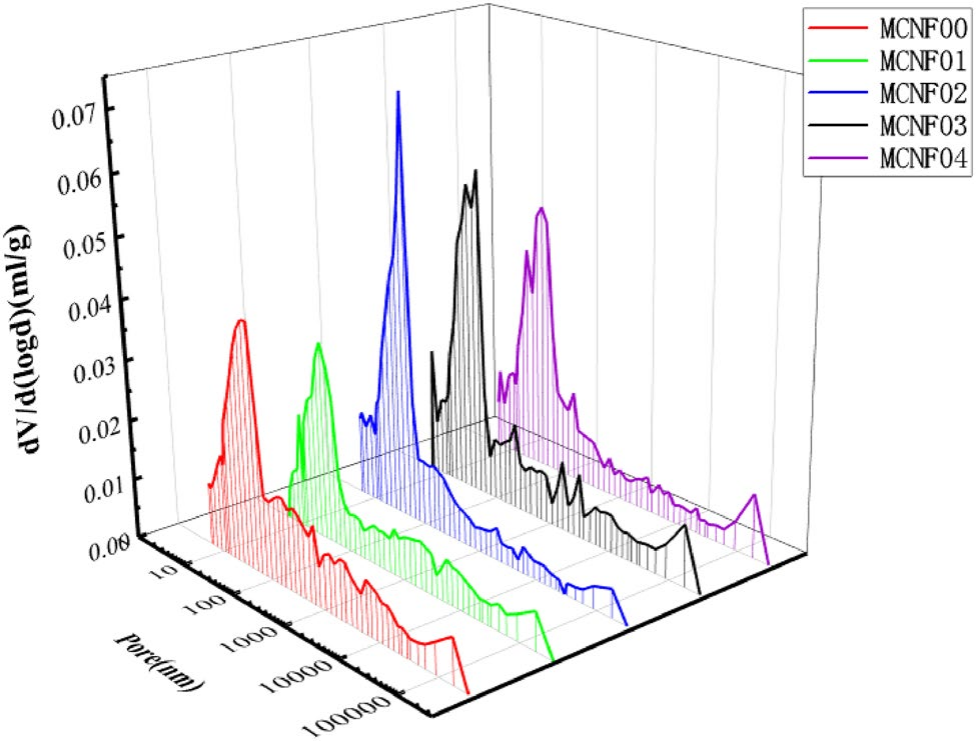
Effects of MCNF on the pore size distribution.

**Figure 10:**
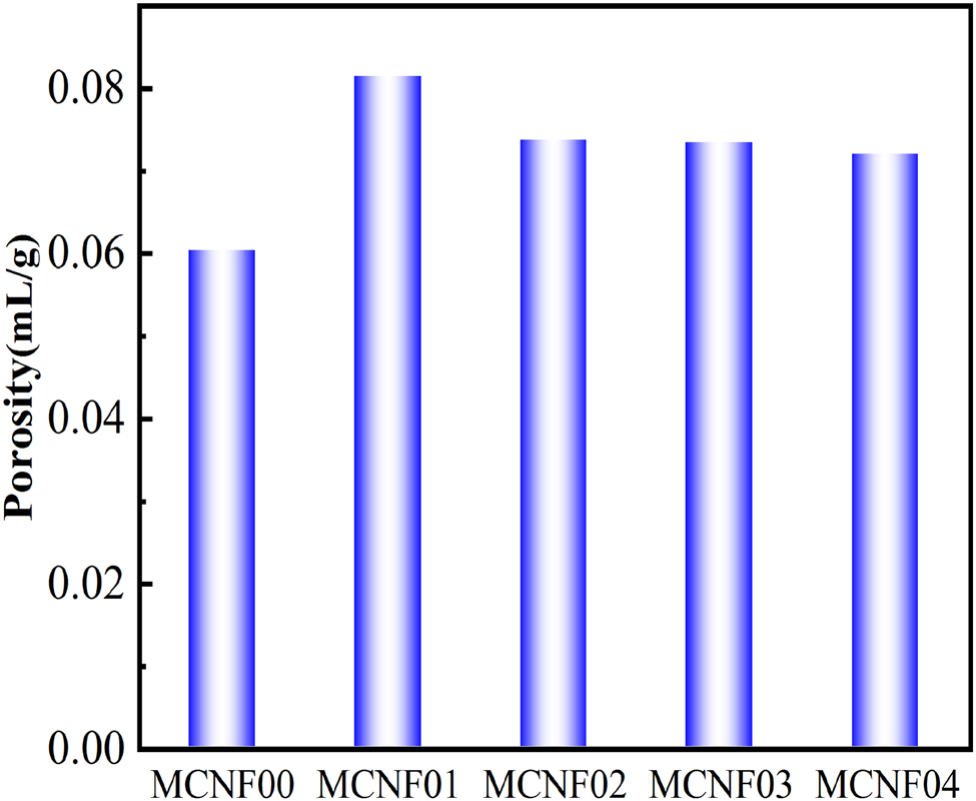
Porosity of MCNF concrete.

The pore size distribution curves and pore proportion distribution curves of the MCNF-reinforced concrete are shown in Figs. 9 and 10. The addition of MCNF ranging from 0.1 to 0.4% significantly improved the porosity of concrete samples by about 0.01–0.02. MCNF promoted sufficient hydration reaction with cement in concrete at the interface, and left larger pores in other parts of the concrete. It is worth noting that too much MCNFs may easily agglomerate, which could only increase the porosity, with limited effect on the mechanical properties of concrete^36^.

**Figure 11:**
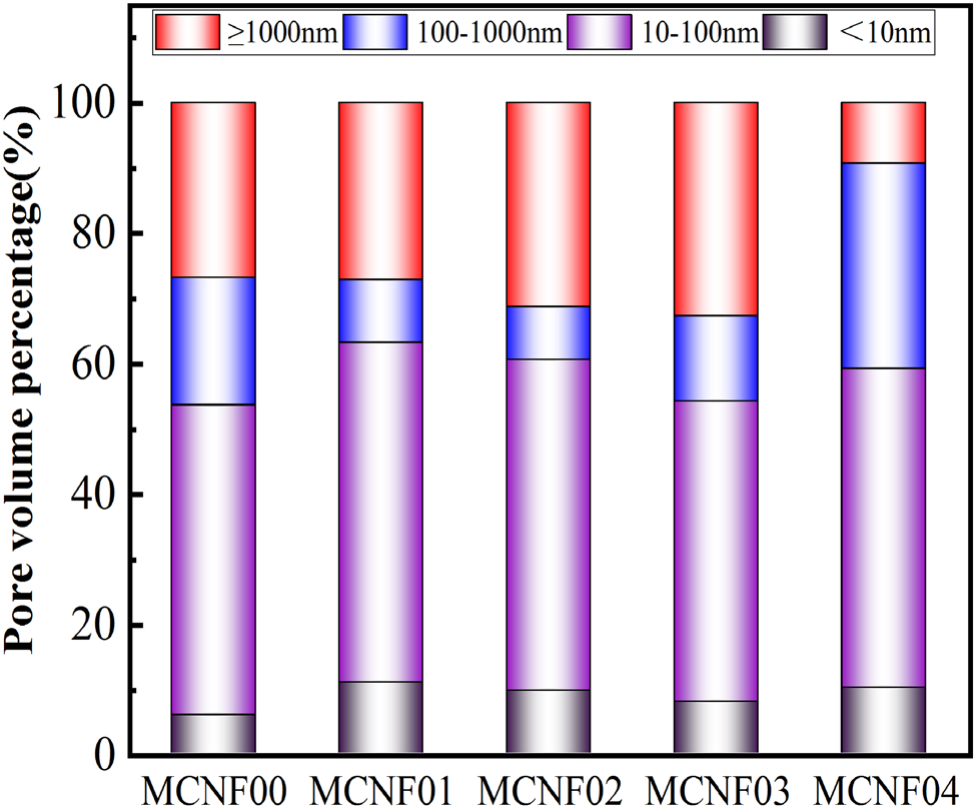
Pore distribution of MCNF concrete.

Pore classification method with reference to ZHANG J X^37^ and pore distribution of MCNF concrete are shown in Fig. 11. The quantity of macropores (≥ 1000 nm) of MCNF concrete increased, transition pores (100–1000 nm) of MCNF concrete decreased, gel pores (10–100 nm) of MCNF concrete and capillary pores (< 10 nm) of MCNF concrete, in most cases, remained unchanged when MCNF content increased from 0 to 0.3%.

The pore size of MCRC is enlarged mainly due to the following three reasons:

- The hydration at the interface between MCNF and concrete is more sufficient because the carbon fiber absorbs the water originally used for mixing during mixing; however, due to the lack of hydration in other parts of concrete, the macropores show an overall trend of enlargement.
- With the increase of the content of MCNF, it becomes increasingly difficult for MCNF to disperse, and the bubbles and pores produced by the matrix gradually increase.
- With the increase of the content of MCNF, it becomes easier for MCNF to aggregate, and the uniform internal force field cannot be formed, resulting in the gradual increase of pores^36^.

**Figure 12:**
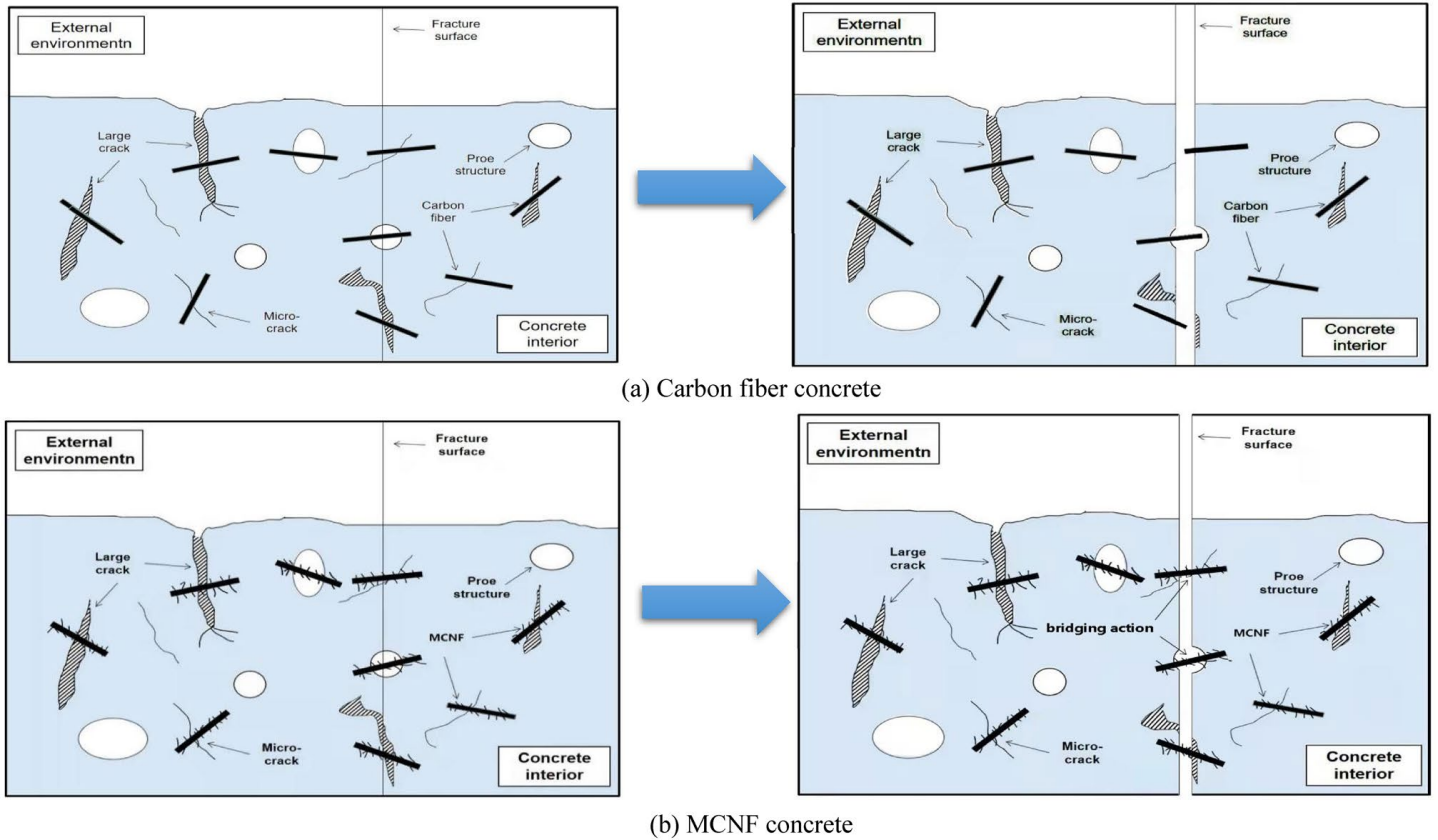
Schematic diagram of failure mechanism of carbon fiber concrete and MCNF concrete.

Combining with the experimental results described above, it can be concluded that the essential reason for the change of dynamic mechanical properties lies in the balance between the porosity and bridging action. Compared with carbon fiber, MCNF has rough surface, which is more difficult to be pulled out, therefore, the bridging effect is more obvious. As shown in Fig. 12b, bridging action refers to that when the concrete is damaged, the carbon fiber in the concrete will connect the two failure surfaces to prevent the development of pores. Compared with ordinary carbon fibers, the surface of MCNF is rougher and the bridging effect is more obvious. The diagrammatic sketch of MCNF and ordinary carbon fibers are shown in Fig. 12a, b.

In the case of low content of MCNF (≤ 0.3%), the dispersion degree in concrete and the porosity will increase less. Therefore, MCNF can effectively connect microscopic cracks in the concrete, thereby improving the mechanical properties of MCNF concrete. In the case that the MCNF content is greater than 0.3%, the pores continue to increase. However, at the same time, due to the large pores, the original equilibrium relationship between pores and bridging is broken, and too much MCNF cannot be effectively dispersed, leading to honeycomb defects in the concrete, the decrease of the bond strength between MCNF and concrete matrix^38^, and the further decline of the mechanical properties of the concrete.

## Engineering application

The dynamic mechanical properties of MCNF concrete are studied. MCNF can improve the dynamic mechanical strength of concrete with low cost, which can be applied, to a certain extent, in the projects with impact load as the main load (such as railway and airport pavement). This study is of reference significance for the improvement of the dynamic mechanical properties of concrete.

## Conclusions

To improve the smoothness of carbon fiber surface, carbon nanotubes were grafted on the surface of carbon fiber by electrophoresis to prepare MCNF. Then, the MCNF concrete was prepared by uniformly mixing MCNF into the concrete. The dynamic mechanical strength of regular concrete and MCNF concrete was studied at different impact speeds, and the modification mechanism was explained. The results are as follows:

1. In CTAB solution, the electrical properties of carbon nanotubes would change, and they can be easily deposited onto the negative electrode.
2. Compared with regular concrete, the dynamic mechanical strength of MCNF concrete increases with the increase of MCNF. It reaches the peak value at the MCNF content of 0.3%.
3. Compared with pure carbon fibers, MCNF has a rougher surface and higher pull-out resistance, resulting in improved dynamic compressive strength.
4. The total pore volume of MCNF concrete increases with the increase of MCNF, and the macropore also increases to a certain extent.

This paper provides a new research idea for the improvement of the surface smoothness of carbon fiber materials.

## Data Availability

The datasets generated and/or analysed during the current study are not publicly available due author's unit does not agree to disclose the data, but are available from the corresponding author on reasonable request.
